# Supplementary material for: Adaptation to host cell environment during experimental evolution of Zika virus
Source: Commun Biol. 2022 Oct 21;5:1115. doi: 10.1038/s42003-022-03902-y (PMC9587232; doi:10.1038/s42003-022-03902-y)
Supplement: Supplementary file 2 — Supplemental Material [file 42003_2022_3902_MOESM2_ESM.pdf]

Supplementary Information

Adaptation to host cell environment during experimental evolution of Zika virus.

Vincent Grass[1], Emilie Hardy[1$], Kassian Kobert[2$], Soheil Rastgou Talemi[3$], Elodie Décembre[1], Coralie Guy[1], Peter V. Markov[2], Alain Kohl[4], Mathilde Paris[5], Anja Böckmann[6], Sara Muñoz-González[1], Lee Sherry[1], Thomas Höfer[3], Bastien Boussau[2]* and Marlène Dreux[1]*

Supplementary note 1, Mathematical model

The multiscale model of ZIKV infection and replication. To have a better understanding of the impact of the delay of the virus replication on ZIKV dynamics we used both intracellular–ZIKV GE·mL^{-1} and extracellular ZIKV measurements–FFU·mL^{-1}. The intracellular dynamics of ZIKV RNA measured in bulk are the sum of replication dynamics in individual cells each of which may have their own time point of infection. To capture this, we construct a multi-scale model of infection, where the relative time course of viral replication is the same in each cell, but the times of infection and, with a delay, of the start of replication, vary. Similar to previous studies on HCV[60,61], we developed a system of coupled partial and ordinary differential equations as follows. Susceptible cells (S) get infected by ZIKV with rate k_{inf}:

$$\frac{dS}{dt} = -k_{inf}\, S\, V \tag{1}$$

where V denotes the concentration of virions. Infected cells I, have an age of infection, a, measuring how much time elapsed since infection. Their number is given by the age-structured balance equation k_{icd}:

$$\frac{\partial I}{\partial t} + \frac{\partial I}{\partial a} = -k_{icd} \cdot I(a,t) \tag{2}$$

which also accounts for cell death with rate k_{icd} which we set to 0.0144 (h^{-1})–assuming a 48 hours half-life for the infected cells [62]. For simplicity, cell proliferation is neglected. We have the initial condition $I(a,0) = 0$ and the boundary condition $I(0,t) = k_{inf} \cdot S \cdot V$ representing the new infections. The dynamics of the virions is obtained by integrating over infected cells of all ages:

$$\frac{dV}{dt} = k_{vp} \cdot \int_{0}^{72\,hrs} I(a,t) \cdot VR(a)\, da - k_{\text{vd}} \cdot V \tag{3}$$

where infectious virions are produced with rate k_{vp} and decay with rate k_{vd}; these parameters were set to 41 (vir·cell^{-1}·h^{-1}) and 0.06 (h^{-1}) respectively [63,64]. Eq. 3 depends on the intracellular ZIKV RNA replication status VR(a), which we describe by a piecewise linear function:

$$VR(a) = \begin{cases} 0 & a < \tau_x \\ k_{rep} \cdot (a - \tau_x) & \tau_x \le a \le \tau_x + \tau_r \cdot \\ 1 & a > \tau_x + \tau_r \end{cases} \tag{4}$$

with τ_{x} being the delay from infection to the onset of virus replication, and τ_{r} the time required to reach maximum replication capacity which we set to 20 hours as measured for Dengue virus [65]. The ZIKV replication rate k_{rep} was set such that the maximum replication capacity is reached 20 hours following virus replication onset ($k_{\text{rep}} = 0.05$ h^{-1}). The delay for the parental virus τ_{p} and the adapted virus τ_{A} are estimated (**Supplementary Fig. 7**). Finally, the number of ZIKV RNA per cells was calculated as:

$$R_{pc}(t) = \frac{\int_0^{72\,hrs} I(a,t) \cdot R(a)\, da}{S(t) + I(t)} \tag{5}$$

where the R(a) is obtained as:

$$R(a) = R_0 + R_{max} \cdot VR(a) \tag{6}$$

with R_0 being the initial number of ZIKV RNA in the cells upon infection, R_{max} the maximum number achieved by replication.

The model was simulated using the method of lines, discretizing first derivatives according to the upwind rule[66]. For this purpose, we used CVODES, a module from the SUNDIALS numerical simulation package, in the MATLAB environment.

To simulate ZIKV transfection experiment, as in Fig. 4c, we assumed that I_0 cells are successfully transfected, which we estimate. Then, the number of virions produced by these cells at 24 hours post-transfection was simulated and used to simulate the number of infected cells at 48 hours post infection upon infection with parental or E S455L mutant virus, consistent with the experimental procedure explained for counting foci (Methods, Analysis of extracellular infectivity). For both the models addressing the delay or the rate hypothesis, parameters k_{inf}, τ_P, τ_A, R_0, R_{max}, and I_0 were estimated while the other parameters were fixed (**Supplementary Fig. 7**). Parameter estimation was conducted by minimizing the weighted least-squares of the simulated values versus the experimental data

$$wSSR = \sum_{i=1}^{N} \sum_{j=1}^{M} \left[\frac{y_{simulation_{i,j}} - y_{experiment_{i,j}}}{\sigma_{i,j}} \right]^2 \tag{7}$$

where j is the experiment number and i is the data point index in time for the jth experiment as:

$$i = \begin{Bmatrix} 6, 24, 48\ (h) & y_{simulation} = R_{pc}(t) \\ 48\ (h) & y_{simulation} = \dfrac{I_{E\ S455L}(t)}{I_{WT}(t)} \end{Bmatrix}$$

$$j = \begin{Bmatrix} RNA\_WT, MOI = 0.01\,, 0.1 \\ RNA\_E\ S455L, MOI = 0.01\,, 0.1 \\ Infected\ cells\ ratio \end{Bmatrix}. \tag{8}$$

The sum of squared residuals is normalized with the standard deviation for each experimental data point $\sigma_{i,j}$. The logarithm of ZIKV RNA and ffu/ml^{-1} values with base 10 was used to estimate model parameters. We calculated the 95% confidence interval for the estimated parameter values using the profile-likelihood method[67] (**Supplementary Fig. 7**). The maximum-likelihood for the remaining parameters is calculated. Finally, the change in the maximum-likelihood ($\Delta\chi^2$) versus different values of the parameter of interest is plotted as profile-likelihood.

References

60 Guedj, J., Dahari, H., Uprichard, S. L. & Perelson, A. S. The hepatitis C virus NS5A inhibitor daclatasvir has a dual mode of action and leads to a new virus half-life estimate. *Expert Rev Gastroenterol Hepatol* 2013, **7**, 397-399.

61 Rong, L. & Perelson, A. S. Mathematical analysis of multiscale models for hepatitis C virus dynamics under therapy with direct-acting antiviral agents. *Math Biosci* 2013, **245**, 22-30.

62 Frumence, E. *et al.* The South Pacific epidemic strain of Zika virus replicates efficiently in human epithelial A549 cells leading to IFN-beta production and apoptosis induction. *Virology* 2016, **493**, 217-226.

63 Best, K. & Perelson, A. S. Mathematical modeling of within-host Zika virus dynamics. *Immunol Rev* 2018, **285**, 81-96.

64 Goo, L. *et al.* Zika Virus Is Not Uniquely Stable at Physiological Temperatures Compared to Other Flaviviruses. *mBio* 2016, **7**.

65 Talemi, S. R. *et al.* Dengue virus is sensitive to inhibition prior to productive replication. *Cell Rep* 2021, **37**, 109801.

66 Shakeri, F. & Dehghani, M. The method of lines for solution of the one-dimensional wave equation subject to an integral conservation condition. *Computers & Mathematics with Applications* 2008, **56**, 2175-2188.

67 Raue, A. *et al.* Structural and practical identifiability analysis of partially observed dynamical models by exploiting the profile likelihood. *Bioinformatics* 2009, **25**, 1923-1929.

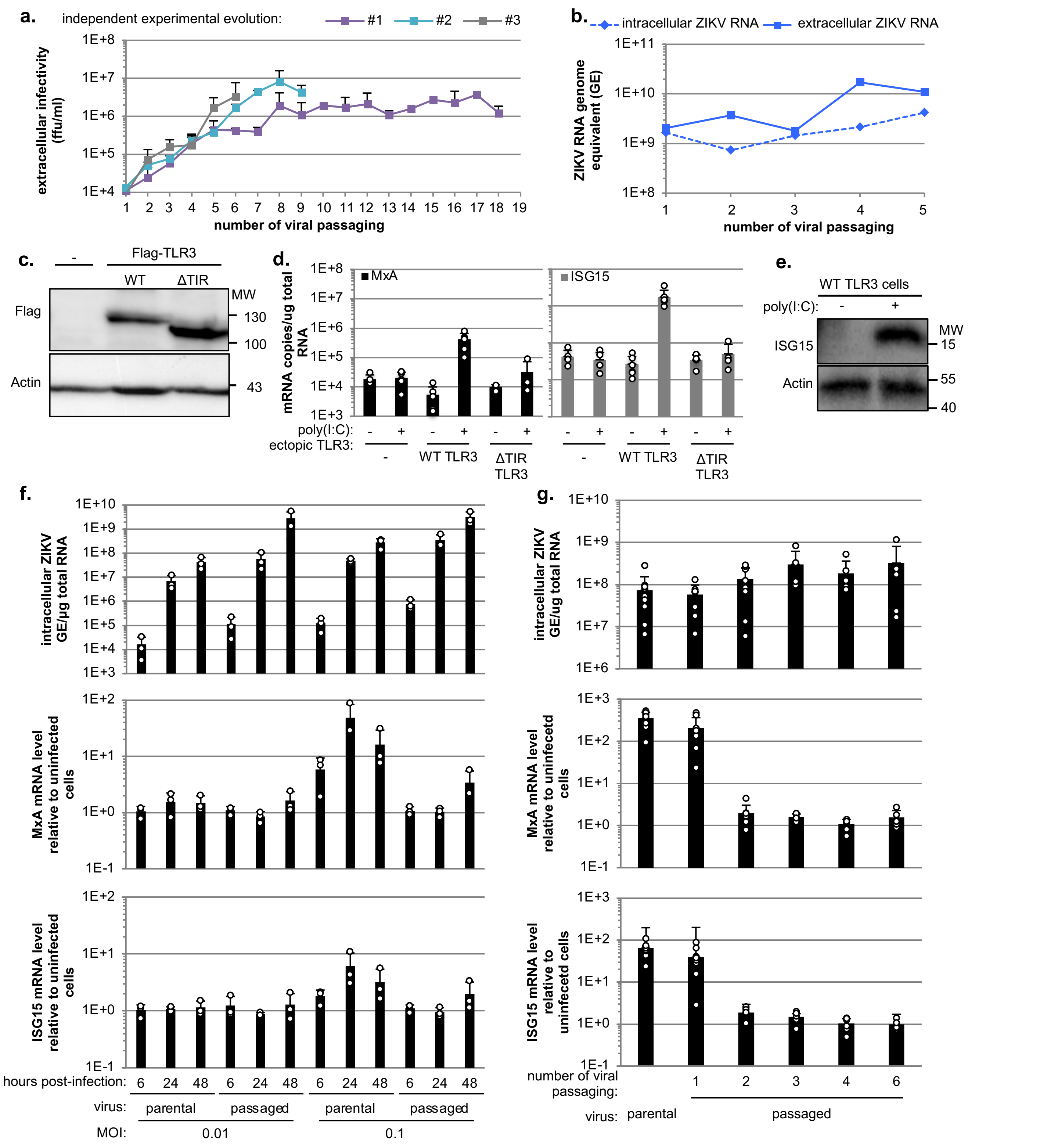
a.
independent experimental evolution:
#1
#2
#3
extracellular infectivity (ffu/ml)
number of viral passaging
b.
intracellular ZIKV RNA
extracellular ZIKV RNA
ZIKV RNA genome equivalent (GE)
number of viral passaging
c.
Flag-TLR3
WT
ΔTIR
MW
Flag
130
100
Actin
43
d.
MxA
ISG15
mRNA copies/ug total RNA
poly(I:C):
ectopic TLR3:
WT TLR3
ΔTIR TLR3
e.
WT TLR3 cells
poly(I:C):
ISG15
Actin
MW
15
55
40
f.
intracellular ZIKV GE/µg total RNA
MxA mRNA level relative to uninfected cells
ISG15 mRNA level relative to uninfected cells
hours post-infection:
virus:
parental
passaged
MOI:
0.01
0.1
g.
intracellular ZIKV GE/ug total RNA
MxA mRNA level relative to uninfecetd cells
ISG15 mRNA level relative to uninfecetd cells
number of viral passaging:
virus:
parental
passaged

Supplementary Fig. 1: Validation of experimental evolution set up, cellular model and analysis of ISG response upon infection by parental virus and passaged viral populations. a Quantification of the extracellular infectivity of the viral populations harvested over serially passaging in the course of 3 independent runs of experimental evolution (referred to as #1, #2, #3; stop at passage 18, 7 and 6, respectively). Results are expressed as foci forming unit (ffu/ml); determined by 2-to-3 independent measurements for each independent run of experimental evolution; mean ± SD. **b** Quantification of extracellular and intracellular ZIKV genome (genome equivalent; GE) levels in serially passaged viral populations by RT-qPCR, results obtained for replicate #1. **c** Western blot analysis of ectopic Flag-tagged WT TLR3 and ΔTIR TLR3 expression in Huh7.5.1 cells, negative control as reference (no ectopic expression; -); actin used as loading control. **d-e** Analysis of MxA and ISG15 induction upon addition of poly(I:C) at 100 µg/mL by quantifying MxA and ISG15 mRNA levels by RT-qPCR (**d**) and western blot (**e**). **f** Quantification of the intracellular ZIKV RNA (upper panel), MxA (middle panel) and ISG15 mRNA (lower panel) levels at the indicated time-points post-infection of Huh7.5.1 cells by parental virus and viral populations harvested at passage 12 of the viral passaging. Infections were performed at MOI 0.1 and 0.01 as indicated; 3 independent experiments; mean ± SD. **g** Quantification of the intracellular ZIKV RNA (upper panel), MxA (middle panel) and ISG56 (lower panel) mRNA levels at 24 hours post-infection by viral populations harvested at the indicated times of the serial passaging viral population and parental virus. Infections performed at MOI 1; 3 independent experiments; mean ± SD.

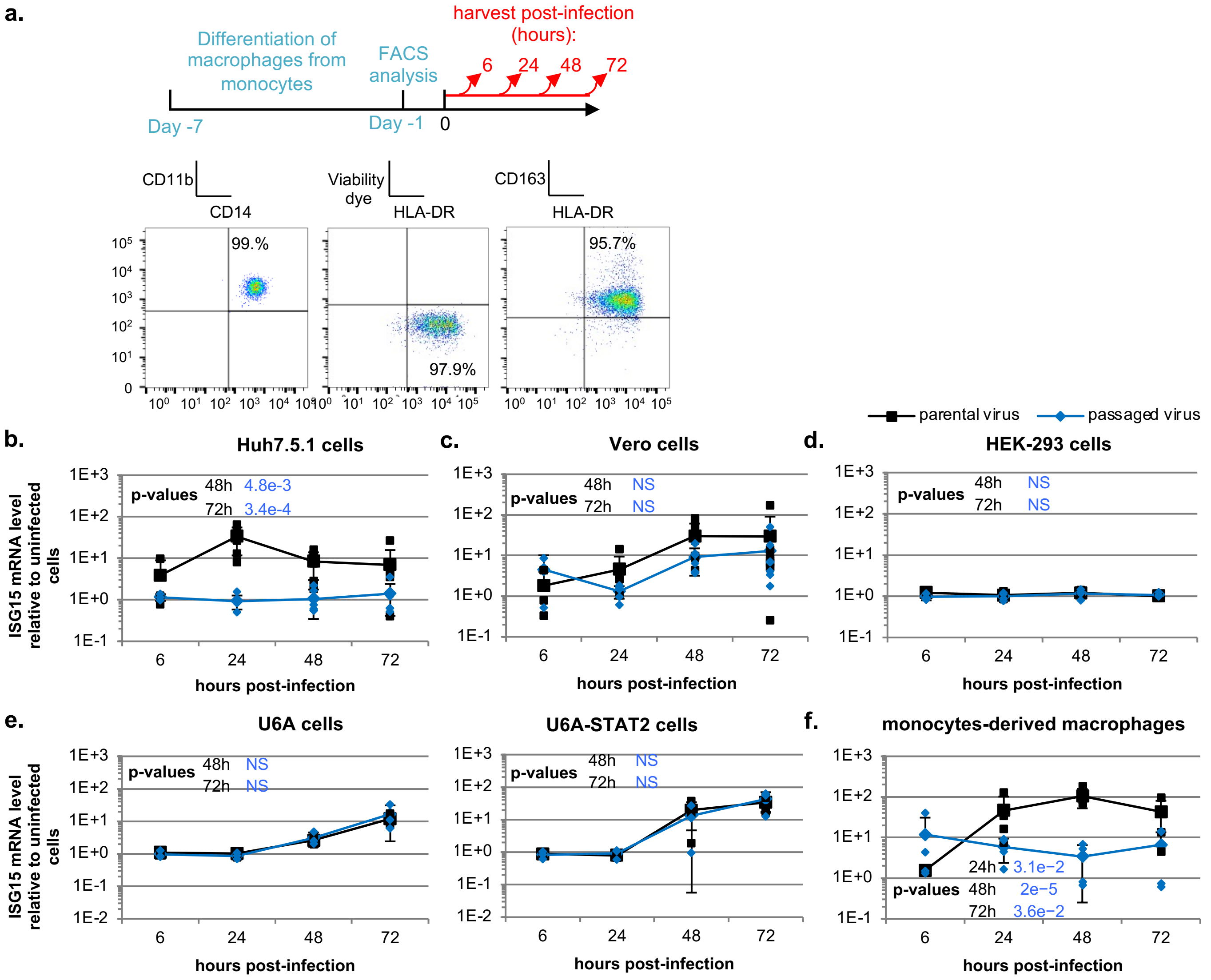

Supplementary Fig. 2: Comparison of viral replication of parental virus and passaged viral population in various cell lines. a Upper panel, schematic representation of the experimental timeline for macrophages derived from monocytes and infection. Lower panels, their differentiation levels were determined by FACS at the day prior infection and using CD11b, CD14, HLA-DR, CD163 differentiation markers and viability dye, as indicated; representative of 4 independent experiments. **b-f** Kinetic quantification of ISG56 mRNA levels relative to the levels in non-infected cells, at the indicated time post-infection at MOI 0.1 by parental virus *versus* serially passaged viral population (*i.e.,* passage 17) in Huh7.5.1 (**b**), Vero cells (**c**), HEK-293 cells (**d**), U6A cells and STAT2 expressing U6A cells [U6A-STAT2 cells] (**e**), and macrophages derived from monocytes (**f**). 3-to-7 independent experiments; mean ± SD. The p-values of the statistical analysis of the kinetics performed using mixed linear model are indicated on the right side of the graphs, p-values are for the comparison of passaged viral population *versus* parental virus, and NS; p>0.05.

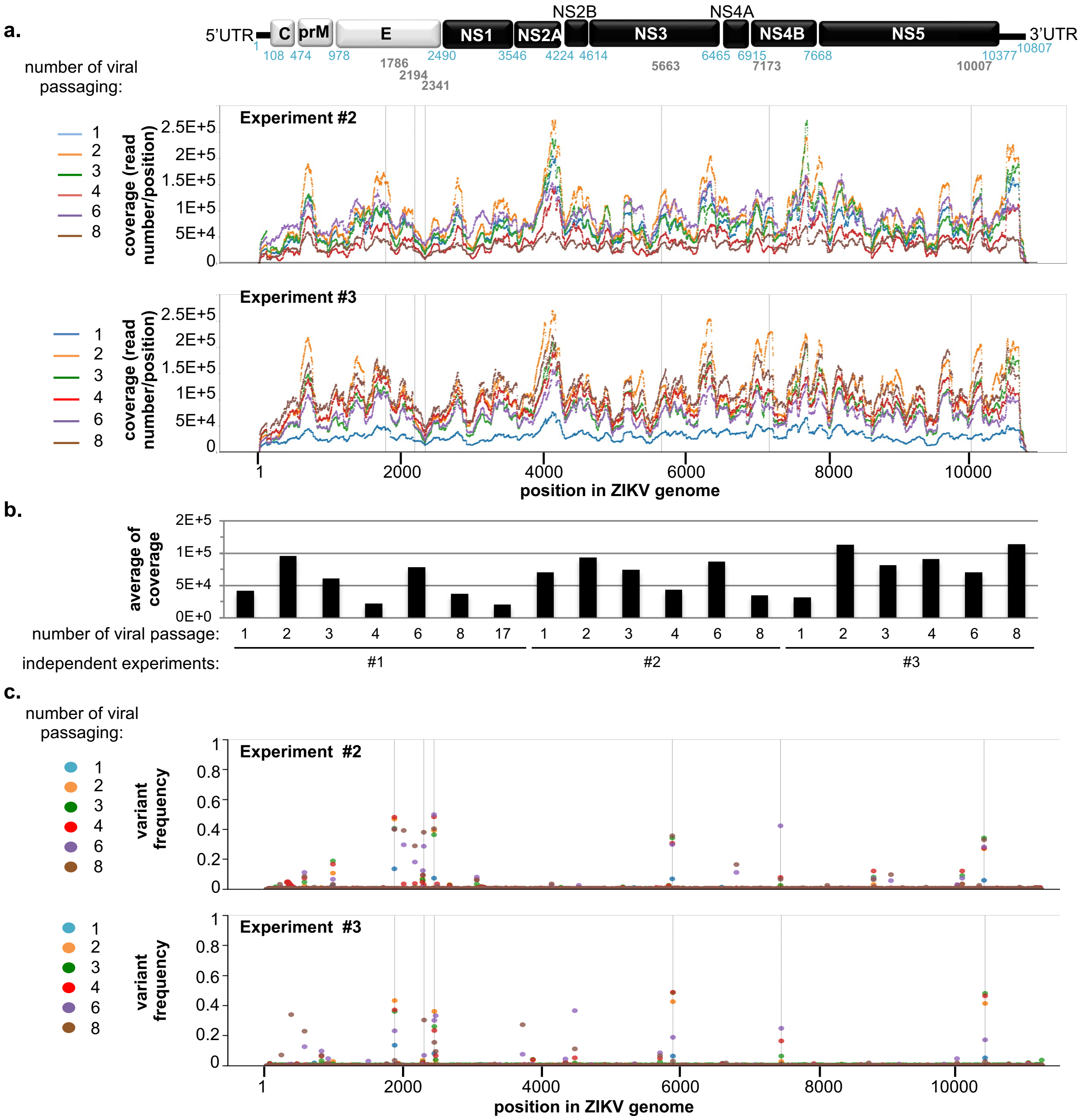

Supplementary Fig. 3: Bioinformatic analysis of the genetic evolution of viral populations obtained by next-generation sequencing. a Sequencing coverage along the ZIKV genome sequence of viral populations harvested at the indicated time points during serial passaging for independent runs of evolution (experiments #2 and #3). Schematic representation of ZIKV genome at the top. **b** Average coverage of the sequenced ZIKV genome for the serially passaged viral population contained in supernatants harvested at the indicated time-points and for independent runs of evolution experiment. **c** Time-course quantification of the frequency of the second most frequent variants at each position along ZIKV genome in the viral populations harvested in independent runs of evolution experiment #2 and #3. Dotted lines indicate the positions in the viral genome with high standard deviations in several runs of experimental evolution, as defined in **Supplementary Fig. 4b-c**.

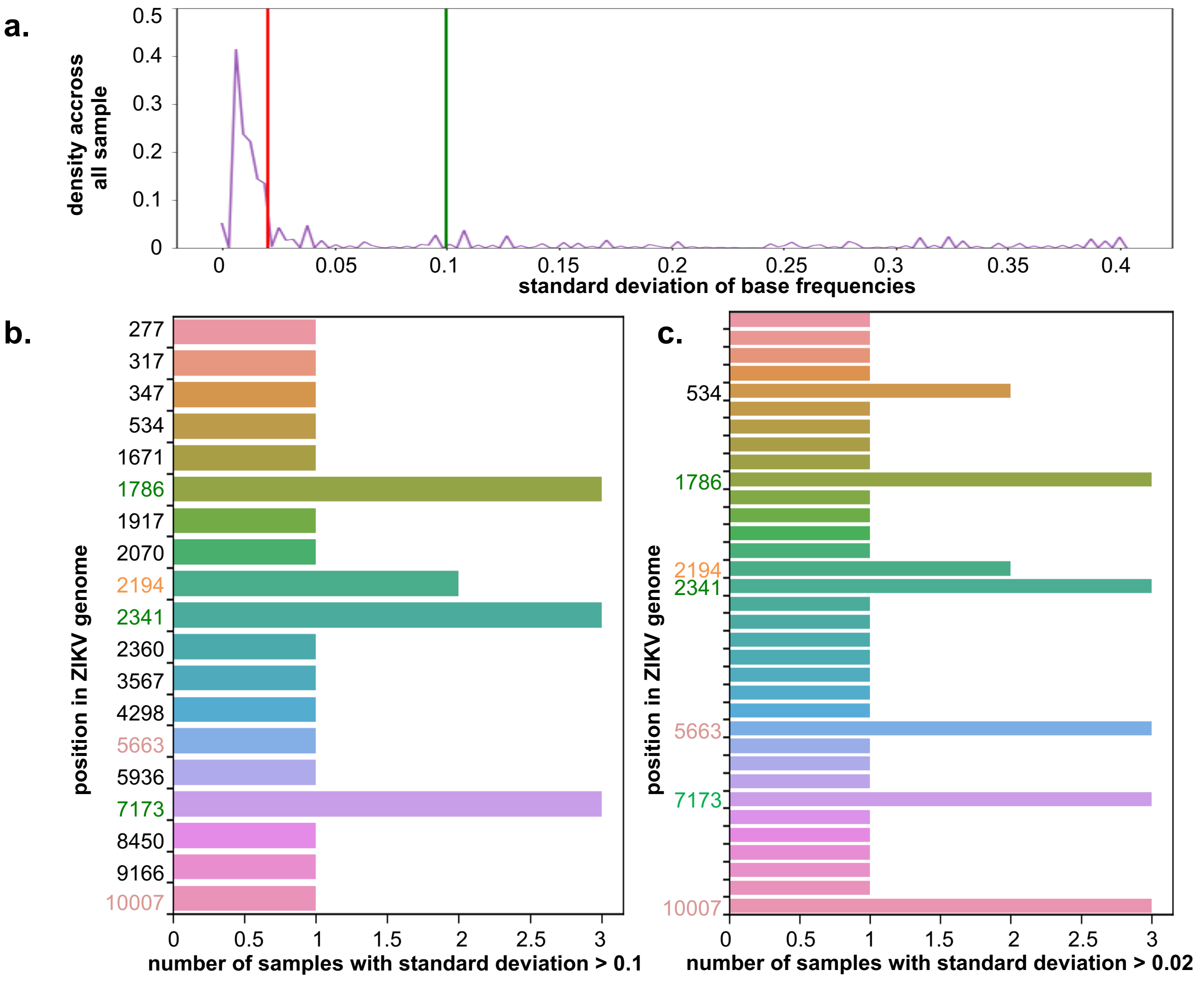

Supplementary Fig. 4: Bioinformatics analysis leading to the selection of candidate adaptive mutations. a Distribution of the standard deviations of variant frequencies across all analyzed samples. Thresholds at 0.02 (red line) and 0.1 (green line) are used for the selection of variants of interest. **b-c** Representation of the number of samples where a given variant is above the standard deviation of 0.1 (**b**) and 0.02 (**c**) across ZIKV genome.

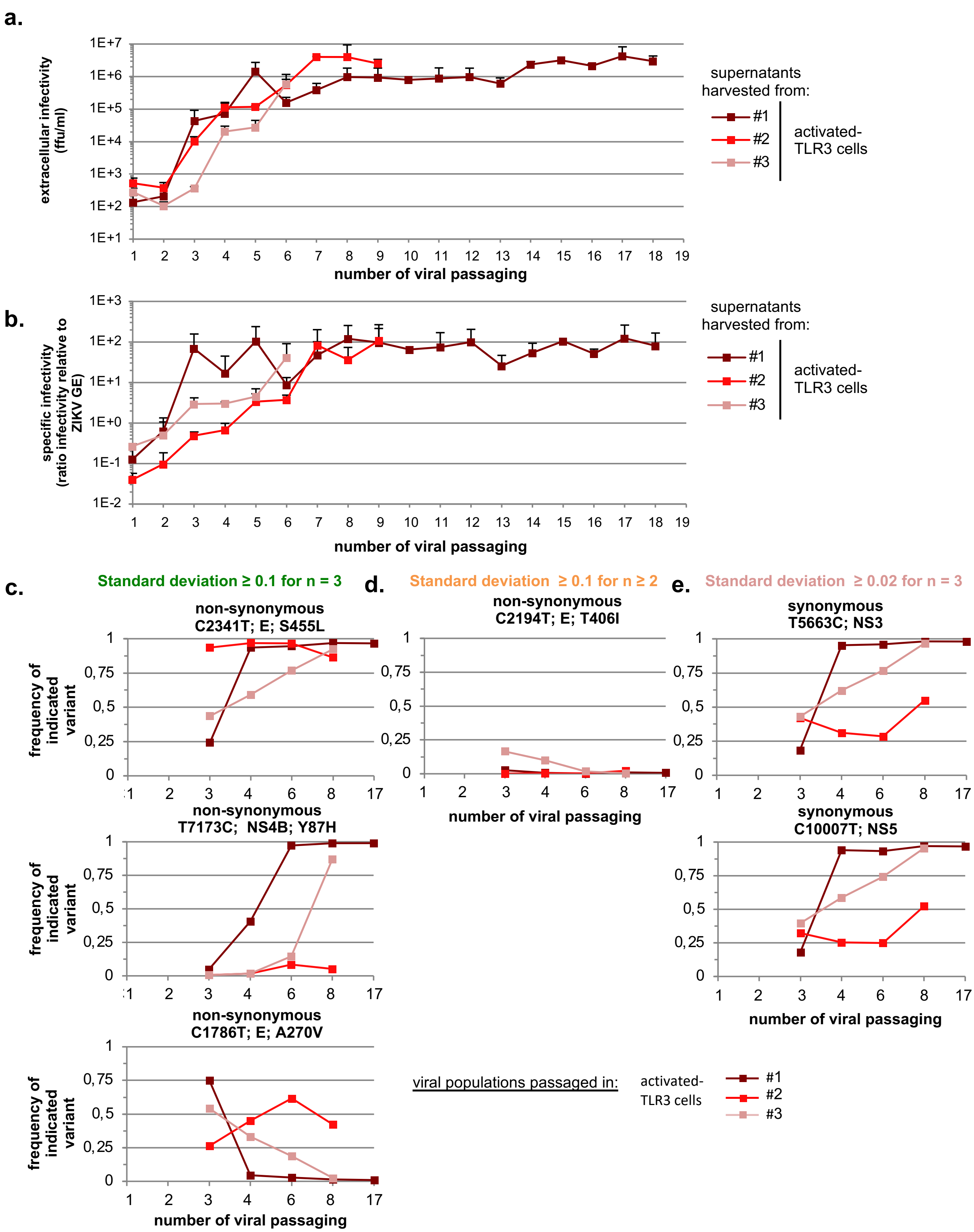
a.
extracellular infectivity (ffu/ml)
number of viral passaging
supernatants harvested from:
#1
#2
#3
activated-TLR3 cells
b.
specific infectivity (ratio infectivity relative to ZIKV GE)
number of viral passaging
supernatants harvested from:
#1
#2
#3
activated-TLR3 cells
c.
Standard deviation ≥ 0.1 for n = 3
non-synonymous C2341T; E; S455L
non-synonymous T7173C; NS4B; Y87H
non-synonymous C1786T; E; A270V
frequency of indicated variant
number of viral passaging
d.
Standard deviation ≥ 0.1 for n ≥ 2
non-synonymous C2194T; E; T406I
number of viral passaging
e.
Standard deviation ≥ 0.02 for n = 3
synonymous T5663C; NS3
synonymous C10007T; NS5
number of viral passaging
viral populations passaged in:
activated-TLR3 cells
#1
#2
#3

Supplementary Fig. 5: Bioinformatic analysis of candidate mutations obtained during experimental evolution with TLR3 activation. a-b Quantification of the extracellular infectivity (**a**) and the specific infectivity (*i.e.,* defined as the probability for one physical virion to initiate infection) (**b**) of the viral populations harvested in activated-TLR3 Huh7.5.1 cells over serially passaging in 3 independent runs of experimental evolution (referred to as #1, #2, #3; stop at passage 18, 7 and 6 respectively). Results are expressed as foci forming unit (ffu/mL) (**a**) and ratio of the extracellular infectivity levels relative to extracellular ZIKV RNA levels and normalized to passage 1 set to 1 (**b**); for each independent passaging; 2-to-3 independent determinations; mean ± SD. **c-e** Time-course quantification of the frequency of variants isolated in Huh7.5.1 activated-TLR3 cells, determined by next-generation sequencing. The variants were selected when the standard deviations of their frequencies were: ≥ 0.1 for all the 3 independent runs of experimental evolution (n =3; n referred to one replicate of one condition at given time of harvest) (**c**); ≥ 0.1 for a minimum of 2 samples (**d**), and ≥ 0.02 for a for all the 3 independent runs of experimental evolution (**e**), with thresholds defined according to the density of variants relative to their frequency for the pool of all analyzed samples, as presented in **Supplementary Fig. 4b-c**. The variants are indicated as nucleotide position (*e.g.,* C2340T), the corresponding viral protein (*e.g*., E) and amino acid change for non-synonymous mutations (*e.g.,* S455L).

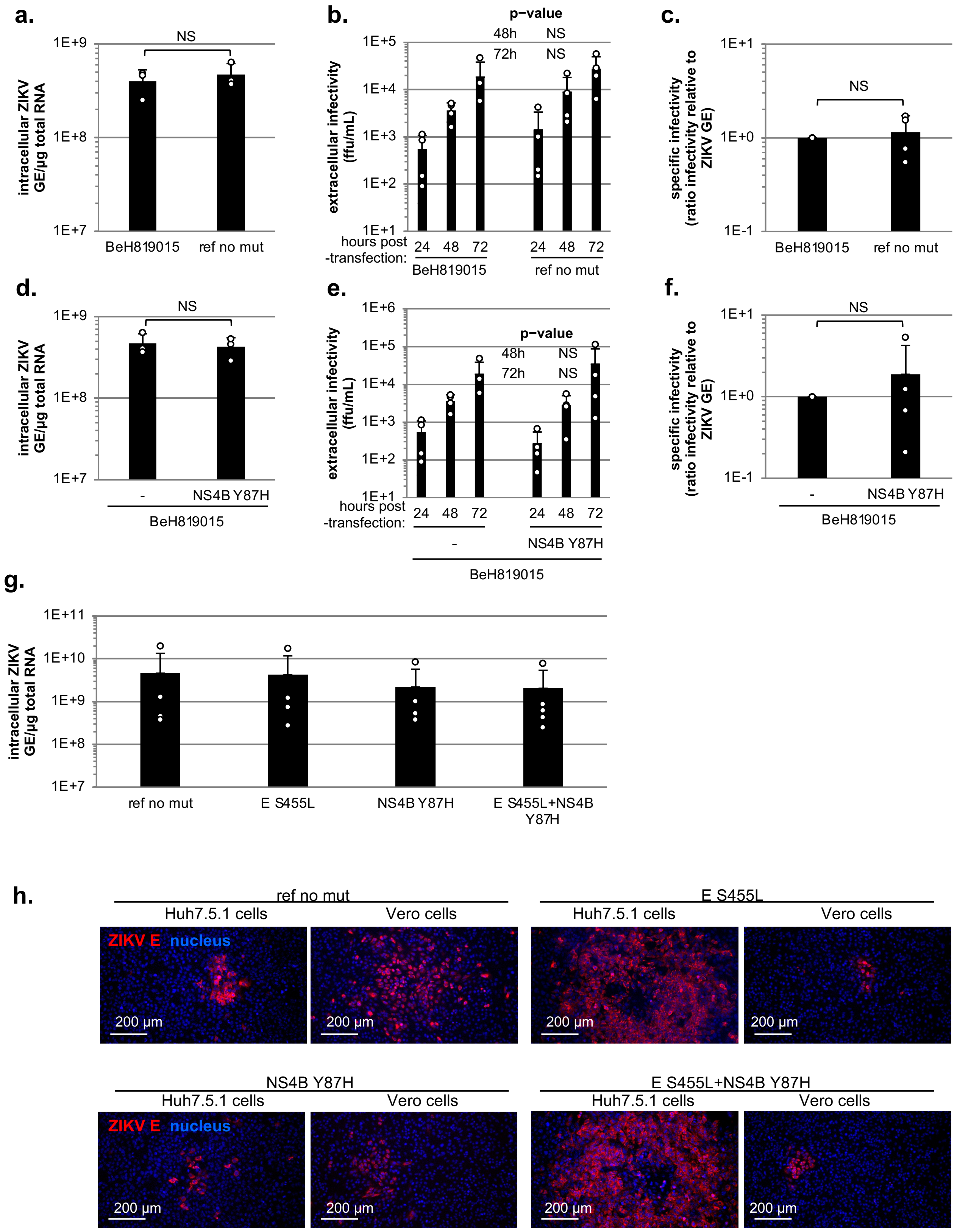

a.
NS
1E+9
1E+8
1E+7
intracellular ZIKV GE/µg total RNA
BeH819015
ref no mut
b.
p-value
48h NS
72h NS
1E+5
1E+4
1E+3
1E+2
1E+1
extracellular infectivity (ffu/mL)
hours post -transfection:
24 48 72
24 48 72
BeH819015
ref no mut
c.
NS
1E+1
1E+0
1E-1
specific infectivity (ratio infectivity relative to ZIKV GE)
BeH819015
ref no mut
d.
NS
1E+9
1E+8
1E+7
intracellular ZIKV GE/µg total RNA
-
NS4B Y87H
BeH819015
e.
p-value
48h NS
72h NS
1E+6
1E+5
1E+4
1E+3
1E+2
1E+1
extracellular infectivity (ffu/mL)
hours post -transfection:
24 48 72
24 48 72
-
NS4B Y87H
BeH819015
f.
NS
1E+1
1E+0
1E-1
specific infectivity (ratio infectivity relative to ZIKV GE)
-
NS4B Y87H
BeH819015
g.
1E+11
1E+10
1E+9
1E+8
1E+7
intracellular ZIKV GE/µg total RNA
ref no mut
E S455L
NS4B Y87H
E S455L+NS4B Y87H
h.
ref no mut
E S455L
Huh7.5.1 cells
Vero cells
ZIKV E nucleus
200 µm
NS4B Y87H
E S455L+NS4B Y87H

Supplementary Fig. 6: Validation of ZIKV molecular clone used for the introduction of the saliant mutations in ZIKV molecular clone. a-c The BeH819015 ZIKV molecular clone (referred to as BeH819015) and derived clones with NS1 mutations, used as reference in **Fig. 4** ("ref no mut") were transfected in Huh7.5.1 cells. **a** Comparable transfection efficiency was assessed by quantification of the intracellular ZIKV GE levels at 6 hours post-transfection; 3 independent experiments; mean ± SD; no statistical difference. **b** Time-course quantification of infectious viral production at the indicated times post-transfection; 4 independent experiments; mean ± SD; no significant difference, as indicated in the table above the graph. **c** Quantification of the specific infectivity in viral supernatants harvested at 72 hours post-transfection of the indicated molecular clones. Results represent the ratio of the extracellular infectivity relative to extracellular ZIKV RNA levels as in **Fig. 1a**. Results present mean ± SD of 4 independent experiments and set to 1 for BeH819015 for each independent analysis. **d-f** The BeH819015 ZIKV molecular clone without mutation (-) *versus* NS4B Y87H mutation introduce in the same background (referred to as BeH819015) were transfected in Huh7.5.1 cells. Results displayed as in panels **a-c;** 3 independent experiments for panel **d** and 4 independent experiments for panels **e-f**; mean ± SD; no statistical difference. **g** ZIKV genome bearing the selected mutations (*i.e.,* single S455L mutation in E, Y87H in NS4B and combined E S455L and NS4B Y87H mutations), and as a reference ZIKV genome without these mutations ("ref no mut"), were transfected in Huh7.5.1 cells. Similar transfection efficiencies were assessed by quantifying the intracellular ZIKV GE levels at 6 hours post-transfection (relative to **Fig. 4a**); 5 independent experiments; mean ± SD; no statistical differences. **h** Representative imaging of infectious foci at 48 hours post-infection of Huh7.5.1 cells using ZIKV bearing the single S455L mutation in E, Y87H in NS4B and combined E S455L and NS4B Y87H mutations, and "ref no mut" as a reference the ZIKV genome without these mutations, performed as in **Fig. 2a**; immunostained E surface proteins (red); nucleus stained by Hoechst (blue); scale-bar indicated.

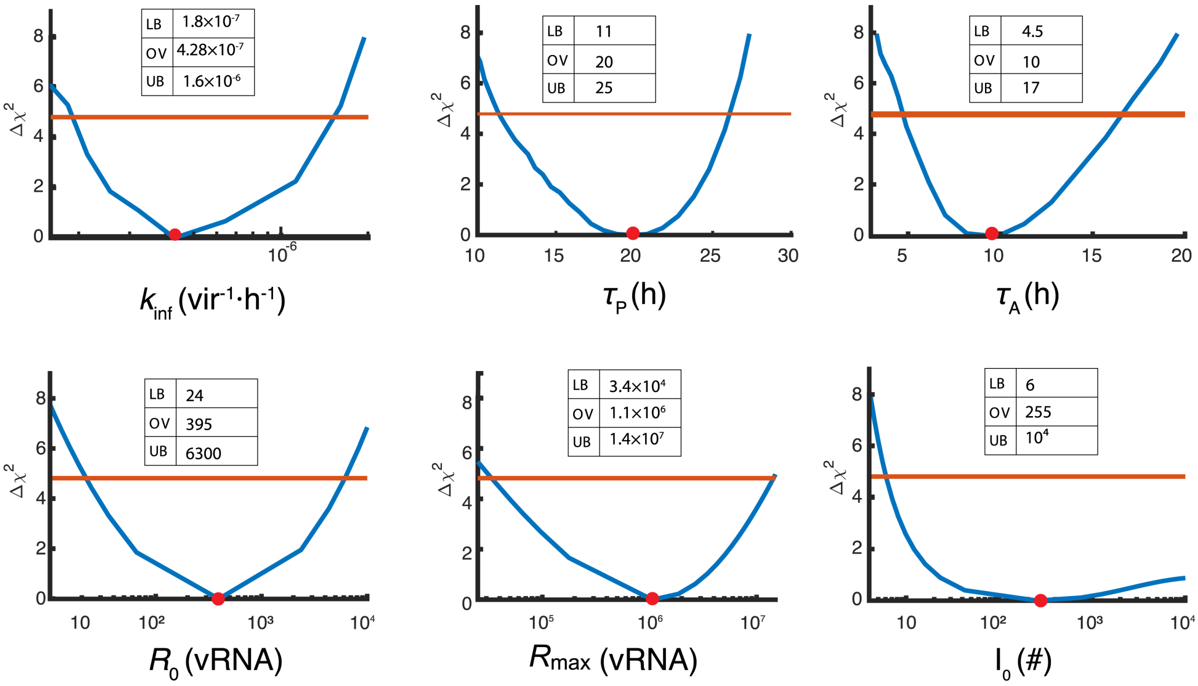

Supplementary Fig. 7: Parameter identification. The 95% confidence intervals for the estimated parameter values were calculated using the profile-likelihood method (**Supplementary Note 1, mathematical model**). The blue curve is the maximum-likelihood profile, the solid red line shows the 95% threshold and the red circle represents the estimated parameter value. The estimated parameter values are reported together with their 95% confidence bounds when the parameter is bounded. Statistic values provided in Table for each graph and as follows: LB; lower bound, OV; optimal value and UB; upper bound of the respective parameters.

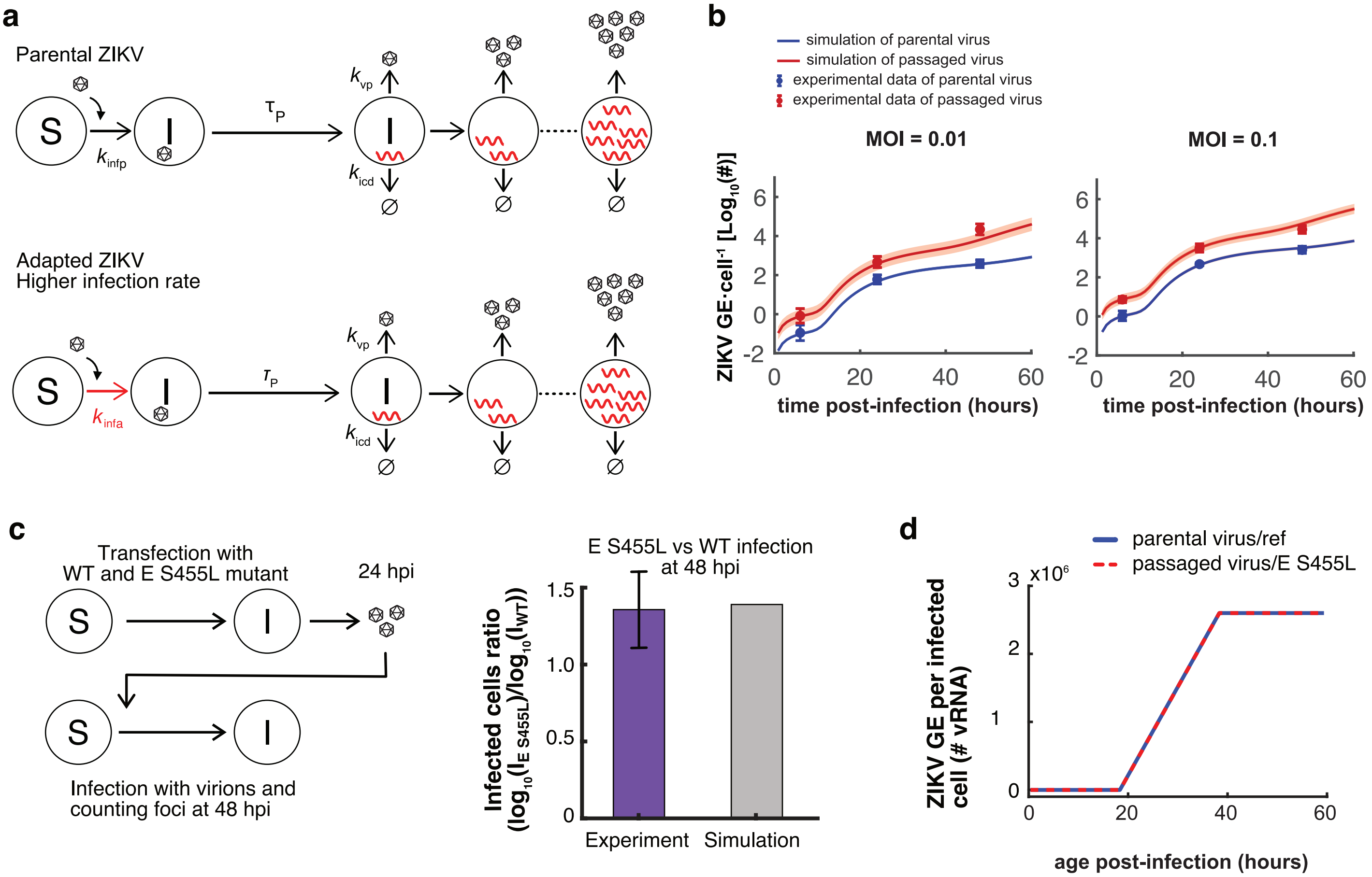

Supplementary Fig. 8: Multiscale model of ZIKV infection and replication, with differences in the infection rate. a Schematic representation of the model. ZIKV infects susceptible cells with rate k_{infp} for the parental virus and k_{infa} for the evolved or mutated viruses. Productive viral replication begins with a time delay τ_{P} for all viruses. Virus replication within infected cells is modeled as a piecewise linear function approximating virus replication (**Supplementary Note 1,** mathematical model). Infected cells produce virions with rate k_{vp}, and die with rate k_{icd}. **b** Model simulation with optimized parameters *versus* experimental measurements of the number of ZIKV genomes per cell upon infection with different doses of parental and adapted ZIKV. **c** Model simulation *versus* experimental measurements of the ratio of the infected cells upon infection with wildtype and the E S455L mutant ($_{\mathrm{E\ S455L}}$ / I_{WT}). The left panel shows the procedure for simulating the transfection experiment consistent with the experimental method for counting foci (**Methods,** Analysis of extracellular infectivity). **d** Simulation of the ZIKV replication inside the infected cells for parental/ref and adapted/E S455L mutant. The estimated delay from infection to viral replication (τ_{P}) is the same for all viral strains.

Relative to Supplementary Fig. 1c.

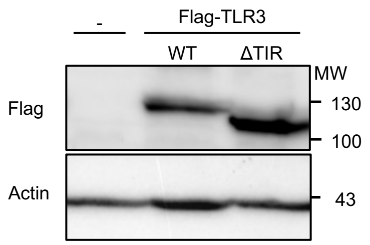

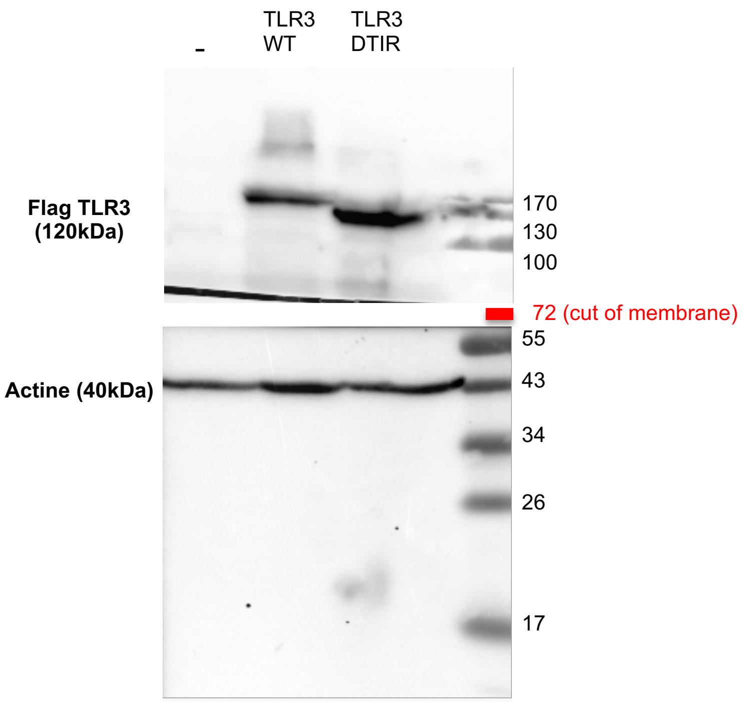

Relative to Supplementary Fig. 1e.

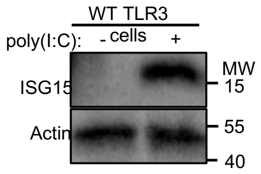

photo of the tranfert membrane to show the molecular weight markers

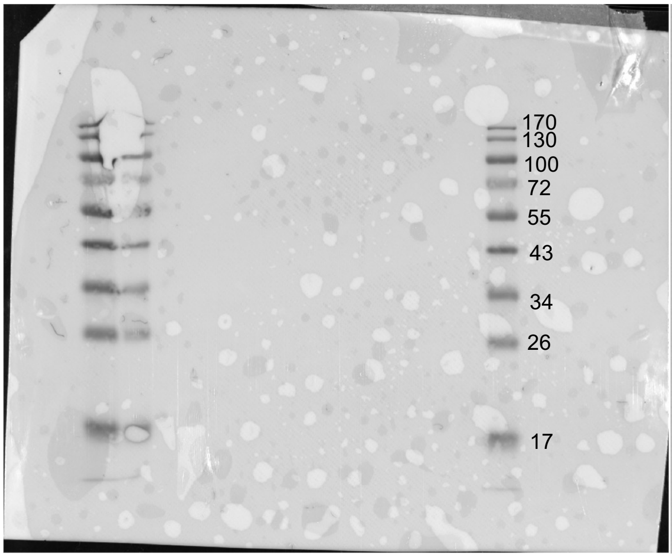

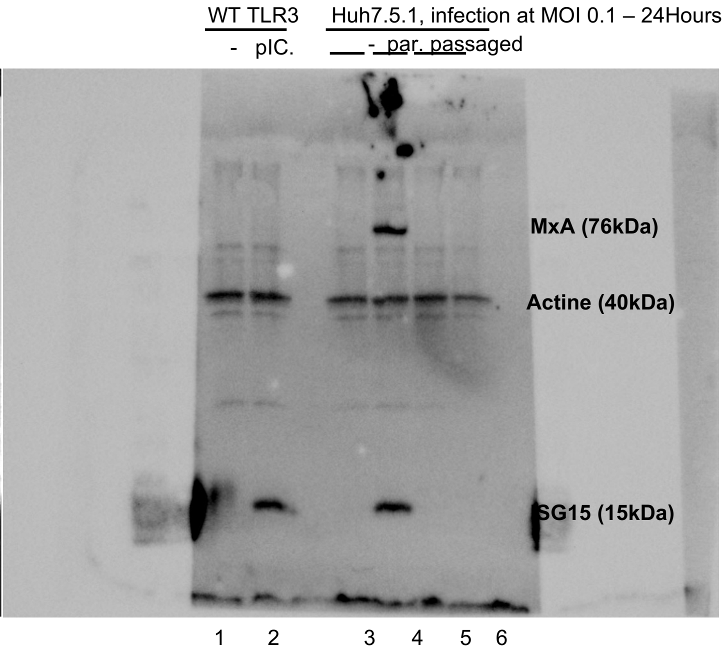

1: WT TLR3 mock
2: WT TLR3 + poly(I:C) at 100 μg/ml for 3hours
3: Huh7.5.1 mock
4: Huh7.5.1 – parental virus (MOI 0.1 for 24hours h)
5: Huh7.5.1 – passaged virus - day 51 (MOI 0.1 for 24hours)
6: Huh7.5.1 – passaged virus - day 51 in activated-TLR3 cells (MOI 0.1 for 24hours)

Supplementary Fig. 9: Initial data scan for the western blot analysis. The results are relative to blot presented in Supplementary Fig. 1c and 1e.

Table S1: Sequences of the primers for RT-qPCR and cloning

**Cloning of ZikV mutants**	
F_Zika_T7172C	GGATGCCATTCCACGCATGGGACTT
Rext_Zika_T7172C	TATTAGAGAGGACGCTGACC
Fext_Zika_T7172C	GGAGTGGGAGTTATGCAAGA
R_ Zika_T7172C	AAGTCCCATGCGTGGAATGGCATCC
F_inf_ZikT7172C	CGTCACAAAAGGATCCGCGCT
R_inf_ZikT7172C	TGCTCACTGCGGATCCTTTCAA
F_Zik_inf_sg	GGGGTTTTGGAAGCCTAGGACTTGATTGTGAACCGAGGAC
R_Zik_inf_sg	GTTCCACATGTTTCCTCCACGTGGACCTTAGTGCCTGGG
**RT-qPCR**	
ZIKV For	ATTGTTGGTGCAACACGACG
ZIKV Rev	CCTAGTGGAATGGGAGGGGA
GAPDH 83U	AGGTGAAGGTCGGAGTCAACG
GAPDH 287L	TGGAAGATGGTGATGGGATTTC
Xef-1a 864U24	ACCAGGCATGGTGGTTACCTTTGC
Xef-1a 970L20	CGACGTTGTCACCGGGCACG
MxA-s	ACAGGACCATCGGAATCTTG
MxA-as	CCCTTCTTCAGGTGGAACAC
ISG15-s	GACAAATGCGACGAACCTCT
ISG15-as	CGGCCCTTGTTATTCCTCA
ISG56-s	GGGCAGACTGGCAGAAG
ISG56-as	CTATAGCGGAAGGGATTTGA
