# Supplementary material for: Adaptation to host cell environment during experimental evolution of Zika virus
Source: Commun Biol. 2022 Oct 21;5:1115. doi: 10.1038/s42003-022-03902-y (PMC9587232; doi:10.1038/s42003-022-03902-y)
Supplement: Supplementary file 3 — Reporting Summary [file 42003_2022_3902_MOESM3_ESM.pdf]

nature portfolio

Corresponding author(s): Bastien Boussau

Last updated by author(s): Feb 16, 2022

Reporting Summary

Nature Portfolio wishes to improve the reproducibility of the work that we publish. This form provides structure for consistency and transparency in reporting. For further information on Nature Portfolio policies, see our Editorial Policies and the Editorial Policy Checklist.

Statistics

For all statistical analyses, confirm that the following items are present in the figure legend, table legend, main text, or Methods section.

n/a	Confirmed	
☐	☒	The exact sample size (*n*) for each experimental group/condition, given as a discrete number and unit of measurement
☐	☒	A statement on whether measurements were taken from distinct samples or whether the same sample was measured repeatedly
☐	☒	The statistical test(s) used AND whether they are one- or two-sided<br>*Only common tests should be described solely by name; describe more complex techniques in the Methods section.*
☐	☒	A description of all covariates tested
☐	☒	A description of any assumptions or corrections, such as tests of normality and adjustment for multiple comparisons
☐	☒	A full description of the statistical parameters including central tendency (e.g. means) or other basic estimates (e.g. regression coefficient) AND variation (e.g. standard deviation) or associated estimates of uncertainty (e.g. confidence intervals)
☐	☒	For null hypothesis testing, the test statistic (e.g. *F*, *t*, *r*) with confidence intervals, effect sizes, degrees of freedom and *P* value noted<br>*Give P values as exact values whenever suitable.*
☐	☒	For Bayesian analysis, information on the choice of priors and Markov chain Monte Carlo settings
☐	☒	For hierarchical and complex designs, identification of the appropriate level for tests and full reporting of outcomes
☐	☒	Estimates of effect sizes (e.g. Cohen's *d*, Pearson's *r*), indicating how they were calculated

Our web collection on statistics for biologists contains articles on many of the points above.

Software and code

Policy information about availability of computer code

Data collection	No software was used for data collection.
Data analysis	Reads were quality-checked and trimmed of sequencing adapters and then mapped using PEAR (https://cme.h-its.org/exelixis/web/software/pear/doc.html) and in-house software (https://github.com/Kobert/viroMapper).<br>A Jupyter notebook reproducing these analyses is available (https://github.com/Boussau/Notebooks/blob/master/Notebooks/Viromics/FiguresAndAnalyses_NoTLR3.ipynb).

For manuscripts utilizing custom algorithms or software that are central to the research but not yet described in published literature, software must be made available to editors and reviewers. We strongly encourage code deposition in a community repository (e.g. GitHub). See the Nature Portfolio guidelines for submitting code & software for further information.

Data

Policy information about availability of data

All manuscripts must include a data availability statement. This statement should provide the following information, where applicable:

- Accession codes, unique identifiers, or web links for publicly available datasets
- A description of any restrictions on data availability
- For clinical datasets or third party data, please ensure that the statement adheres to our policy

Datasets for the sequence data file available in SRA with accession numbers SRX9704326-SRX9704344; bioproject PRJNA686429.

Field-specific reporting

Please select the one below that is the best fit for your research. If you are not sure, read the appropriate sections before making your selection.

☒ Life sciences ☐ Behavioural & social sciences ☐ Ecological, evolutionary & environmental sciences

For a reference copy of the document with all sections, see nature.com/documents/nr-reporting-summary-flat.pdf

Life sciences study design

All studies must disclose on these points even when the disclosure is negative.

Sample size	Evolutionary experiments were conducted in triplicate, and runs 2 and 3 were stopped when viral production reached the level at which run #1 plateaued. This amount of data was found to be enough to recover substitutions that had increased in frequency independently in the three runs.
Data exclusions	No data was excluded from the analysis.
Replication	We used a minimum of three biological experiments were performed independently to assess the reproducibility of our results. All replications were successful.
Randomization	N/A. No randomization was used given the small number of samples and the lack of influence of randomization on the experimental design and experimental approach used. (no animal experiments were performed in this study).
Blinding	The replicates were made using the same conditions. For the computational analysis of the evolutionary experiments, blinding was not used, but the bioinformaticians running the analyses had not made the experiments and therefore had no prior knowledge of possible differences between the runs.

Reporting for specific materials, systems and methods

We require information from authors about some types of materials, experimental systems and methods used in many studies. Here, indicate whether each material, system or method listed is relevant to your study. If you are not sure if a list item applies to your research, read the appropriate section before selecting a response.

Materials & experimental systems

n/a	Involved in the study	
☐	☒	Antibodies
☐	☒	Eukaryotic cell lines
☒	☐	Palaeontology and archaeology
☒	☐	Animals and other organisms
☒	☐	Human research participants
☒	☐	Clinical data
☒	☐	Dual use research of concern

Methods

n/a	Involved in the study	
☒	☐	ChIP-seq
☐	☒	Flow cytometry
☒	☐	MRI-based neuroimaging

Antibodies

Antibodies used	mouse anti-E glycoprotein (4G2) kindly provided by P. Despres (PIMIT, Université de La Réunion-INSERM France) and anti-Mouse IgG (H+L) Alexa Fluor 555 Secondary Antibody (Life Technologies). The antibodies used for western blot detection were anti-ISG15 (Santa Cruz), anti-flag (clone M2; Sigma-Aldrich) and mouse anti-actin (clone AC74; Sigma-Aldrich). The antibodies used for FACS analysis were APC-conjugated anti-CD163 (clone REA812, MACS Miltenyi Biotec), PE-conjugated mouse anti-HLA-DR (clone LN3, Invitrogen); PE-Cy7-conjugated mouse anti-CD11b (clone ICRF44, BD Biosciences), Pacific Blue-conjugated mouse anti-CD14 (clone 63D3, Biolegend).
Validation	Antibodies were validated by over-expression (anti-flag), polyI:C treatment (anti-ISG15) or infection experiments in this study (anti-E glycoprotein). Additionally, antibodies have been validated by the manufacturers.

Eukaryotic cell lines

Policy information about cell lines

Cell line source(s)	Huh-7.5.1 cells from F.V. Chisari (Scripps Research Institute, La Jolla, CA) ; Vero E6 cells from Dr M Bouloy (Institut Pasteur, Paris, France) ; U6A cells from Dr M. Köster (Helmholtz-Zentrum für Infektionsforschung)
Authentication	The identity of all the immortalized cell lines used in this study was confirmed by the providers. Official certification can be

Authentication	provided upon request.
Mycoplasma contamination	All cell lines were tested to be mycoplasma free by standard PCR-based assay.
Commonly misidentified lines (See ICLAC register)	No commonly misidentified cell line was used in this study.

Flow Cytometry

Plots

Confirm that:

☒ The axis labels state the marker and fluorochrome used (e.g. CD4-FITC).

☒ The axis scales are clearly visible. Include numbers along axes only for bottom left plot of group (a 'group' is an analysis of identical markers).

☒ All plots are contour plots with outliers or pseudocolor plots.

☒ A numerical value for number of cells or percentage (with statistics) is provided.

Methodology

Sample preparation	Surface immunostainings were performed at day 6 post-isolation of the CD14+ cells from PBMCs. After a 15 minute-incubation step with human Fc blocking reagent (MACS Miltenyi Biotec), cell surface markers were detected by a 30 minute-incubation at 4°C with 4 µg/mL Pacific Blue-conjugated mouse anti-CD14 (clone 63D3, Biolegend), a 1:20 dilution of APC-conjugated mouse anti-CD163 (clone REA812, MACS Miltenyi Biotec), 0.14 µg/mL PE-conjugated mouse anti-HLA-DR (clone LN3, Invitrogen) and a 1:20 dilution of PE-Cy7-conjugated mouse anti-CD11b (clone ICRF44, BD Biosciences), diluted in staining buffer (PBS - 1% FBS).
Instrument	Flow cytometric analysis was performed using a BD FACS Canto II.
Software	The data were analyzed with Flow Jo software (Tree Star).
Cell population abundance	*Describe the abundance of the relevant cell populations within post-sort fractions, providing details on the purity of the samples and how it was determined.*
Gating strategy	*Describe the gating strategy used for all relevant experiments, specifying the preliminary FSC/SSC gates of the starting cell population, indicating where boundaries between "positive" and "negative" staining cell populations are defined.*

☐ Tick this box to confirm that a figure exemplifying the gating strategy is provided in the Supplementary Information.

March 2021
